# Striking Instance of the Salutary Effects of Vaccination

**Published:** 1808-10

**Authors:** P. Speckman

**Affiliations:** Minister of the Gospel at *Elde*. Elde


### *Striking Instance of the salutary Effects of Vaccination.* Communicated by A. V. H. Tellegen, M. D.

ALTHOUGH we have reason to pride ourselves on the success Vaccination has experienced in this country, it nevertheless appears, that now and then some few children fall victims to the natural small-pox; that such unfortunate objects should attract our notice, and induce us to shed shed a tear of pity, is very natural, especially when it is considered that the negligence of their parents was certainly instrumental to their death; but, however deplorable this state of things may be, I can, however, by no means concur in the assertion that this negligence proceeds from a culpable prejudice against vaccination; for my own part, I am rather induced to suppose, that the omitting of vaccine inoculation, is more to be ascribed to a want of conviction, and, consequently, to ignorance, than to mere vulgar prejudice.

As examples are always more persuasive and efficient than arguments or moral strictures, I thought it would not be unserviceable to make the public acquainted with some facts, which, in my course of practice, have come immediately to my own knowledge, whence the public may judge how far vaccination is entitled to general patronage and adoption. After having, in the course of last year, inoculated about twenty-five children at *Elde* and *Paterswalde*, in the department of *Drenthe*, with vaccine matter, the natural small-pox made its appearance last winter at both these places as usual; they soon became extremely virulent, several children suffered severely, three died of the distemper, and one lost its right eye, while the above mentioned twenty-five children who were vaccinated, remained free from all contagion during the whole of the time that this disease raged there with such virulence; this circumstance naturally attracted the notice of the inhabitants; many parents were induced to have recourse to vaccination, and in the course of a fortnight, one hundred and fifty children of different ages were brought to me for that purpose, and the consequence happily was, that the further progress of the natural small-pox, which threatened the most serious and fatal effects, was not only immediately arrested, but even, (if I may be allowed the expression) so completely exterminated, that since that period, not one child has died, or otherwise suffered by this dreadful disorder. In order to shew that I have not overrated its beneficial effects, I here subjoin the following certificate.

I do hereby certify, that the above number of children belonging to the districts of *Elde* and *Paterswalde*, and in which number I may also include one of my own, have been vaccinated by A. V. H. *Tellegen*, M. D. at *Groningen*, with that happy and salutary effect, that they have thereby entirely escaped the contagion of the natural small-pox which which raged here with so much violence during the course of last winter.

P. SPECKMAN,

*Elde, June* 6, 1808. Minister of the Gospel at *Elde.*

---

" The proofs which this city, (Groningen) affords of the happy effects and power of this inoculation are of no less importance. The number of persons inoculated with vaccine matter may be fairly estimated at about 3,000, many of them were afterwards again inoculated with the small-pox ; others have eaten, slept, and have been continually kept with children who actually at the time had the natural small-pox, without any one of them having hitherto caught the infection. Since two years, the natural small-pox does sometimes make its appearance in some parts of this city, but the disorder is wholly confined to such persons as have hitherto not received the benefit of vaccination. As a proof of the truth of this treatment, I shall only mention, that hitherto no person has applied to me for the premium, (ten ducats) which I two years ago offered for every child, who, after having regularly gone thro' the process of vaccination, should afterwards take the natural small-pox, or should have died in consequence of the vaccine inoculation. I take this opportunity to repeat the offer I made at that time, provided sufficient proofs are given that *such child or children were regularly vaccinated in this city by a person well acquainted with the process of vaccination, and that such person does certify that the pustule proceeding therefrom, was in every respect genuine.*

Heaven grant that every true philanthropist, and especially every magistrate, minister, or school-master, as well in town as in country, would use his influence to recommend and give every degree of publicity to this invaluable preventive, revealed as it were by Heaven for the benefit of the human species; how many parents and children would not thereby be preserved to society!

*Dutch Royal Courant, July* 21, 1808."

